# Fathers’ perceptions of factors associated with the attainment of paternity leave: a qualitative study

**DOI:** 10.3389/fgwh.2024.1466227

**Published:** 2025-01-28

**Authors:** Naoyuki Mita, Yu Par Khin, Nobutoshi Nawa, Yui Yamaoka, Takeo Fujiwara

**Affiliations:** ^1^ Master of Public Health in Global Health (MPH) Course, Institute of Science Tokyo, Tokyo, Japan; ^2^ Department of Public Health, Institute of Science Tokyo, Tokyo, Japan

**Keywords:** paternity leave, factor, qualitative, thematic analysis, Japan

## Abstract

**Background:**

A systematic review has reported that paternal involvement is beneficial in reducing social, behavioural and psychological problems in children. However, little research has investigated factors associated with the attainment of paternity leave.

**Objective:**

The objective of this study was to explore fathers’ perceptions of the factors associated with taking paternity leave.

**Participants and setting:**

Overall, 21 in-depth interviews were conducted with fathers to obtain information on their perceptions of paternity leave. We also conducted key informant interviews with four supervisors at companies to understand their opinions on paternity leaves and ensure that the implications of this study are relevant at workplaces.

**Methods:**

Thematic analysis was adopted to analyse the data. Deductive coding was conducted based on previous studies on paternity leave, followed by inductive coding and modification of the initial codes. The final codes were organised based on the social-ecological model.

**Results:**

Themes associated with taking paternity leave included fathers’ career aspirations, fathers’ commitment to supporting infants or mothers, mothers’ or infants’ need for fathers’ involvement, colleagues’ outlook on paternity leaves, fathers’ work schedules or responsibilities, support from relatives, paternity leave policies, and trends toward fathers’ involvement. The supervisors mentioned themes related to paternity leave from the broader perspective of long-term effects and impact on the organisation.

**Conclusions:**

This study revealed fathers’ perceptions regarding the factors associated with taking paternity leave. The results may provide useful information on how to create an environment in which fathers can easily take paternity leave.

## Introduction

Paternity leave is generally defined as “a short period of leave immediately after childbirth” and aims to “enable fathers to assist the mother to recover from childbirth” (1). It also allows fathers to “develop parenting skills and a sense of responsibility” and “become active parents” (2). A systematic review reported that paternal involvement in childcare reduced behavioral and psychological problems in children while improving their cognitive development (3). Due to these benefits, the number of countries with paternity leave provisions increased from 40 in 1994 to 79 in 2013, though significant variations remain in duration, eligibility criteria, and cash benefits (1). By 2022, this number had risen to 117 countries offering paid paternity leave (4).

### Literature review

Japan introduced paternity leave legislation as early as 1991, allowing fathers to take up to 12 months of paid leave before their child's first birthday (5). Despite this provision, the uptake of paternity leave among Japanese men remained significantly low until 2017, when political reforms were introduced to provide more flexible paternity leave (5). Currently, Japan offers the second-longest total duration of paternity leave among the Organisation for Economic Co-operation and Development (OECD) countries, however uptake remains low, with only 14% of eligible fathers utilizing the benefit in 2021 (6). In contrast, countries like Sweden and Finland reported moderate to high rates of paternity leave uptake, reaching up to 80% in 2021. Paternity leave legislations of these countries share common features such as relatively long leave durations, high financial allowances, and high flexibility, encouraging greater participation (7). Similarly, countries like Spain and Estonia have experienced a rapid increase in paternity leave uptake, reaching around 70% in 2021, following political reforms that extended leave durations and improved financial support (6).

The low uptake of paternity leave in Japan, despite legislative reforms, suggests the presence of persistent barriers. These barriers can be examined using the social-ecological model, which highlights multiple levels of determinants on behavior. These levels include the individual level, with factors such as age, education, and income; the relationship level, involving peers, partners, and family; the community or organizational level, such as schools or workplaces; and societal factors, including political and cultural determinants (8). Previous studies in Europe and the United States have applied the social-ecological model to identify key factors influencing fathers’ use of paternity leave at different levels (9–12). At the individual level, employment and financial stability (10, 11) as well as prioritization of childcare responsibilities (12) are significant determinants. At the relationship level, the employment status of a partner also plays a critical role (10). Organizational and cultural barriers are particularly prominent in male-dominated professions, such as law, where workplace policies and support are crucial for fathers to take paternity leave (9, 11).

Despite extensive research in western countries, studies examining fathers’ perceptions of paternity leave in Japan remain limited. This gap may also be due to gender norms that disproportionately place the childcare burden on women, and Japan's collectivist work culture, which often discourages deviations from traditional norms (13). A brief pilot survey conducted by an international research team in 2024 (14) and a study conducted in 2017 highlighted that cultural norms and workplace barriers were primary reasons why fathers chose not to take paternity leave (15). However, these studies did not comprehensively address determinants of fathers’ perception of paternity leave across different levels as outlined in the social-ecological model. To fill this gap in the literature, our study intends to explore fathers’ perceptions of the factors associated with taking paternity leave in Japan, by applying the social-ecological model.

## Methods

### Data collection

We conducted 21 in-depth online interviews with fathers between September 18, 2021 and October 23, 2021 to obtain information on their perceptions of paternity leave. A sample size of approximately 15 participants is deemed sufficient for qualitative analysis of interview data addressing a specific research question (16). The data collection was stopped upon the attainment of data saturation, defined as a point at which further interviews no longer generated new information (17). We also conducted key informant interviews with four supervisors at companies between September 30, 2021 and October 22, 2021 to understand their opinions on paternity leaves and ensure that the implications of this study are relevant at workplaces. The interviews with company supervisors were designed as key informant interviews with a smaller sample size and were analyzed separately from the more extensive interviews conducted with fathers (18). However, in the discussion section, findings from the fathers’ interviews were integrated with insights from the company supervisors to ensure that the implications of this study are meaningful and relevant in workplace settings. The first author (NM) contacted the initial participants (18 fathers and two company supervisors) through a personal social network. The remaining participants were contacted through early participant referral by four of the initial participants. We contacted participants via social network services or email.

Interviews were conducted in Japanese, and we used separate semi-structured interview guides for 21 fathers and four company supervisors (Supplementary Material). Our interview guide for fathers included questions on their age, occupation, education, children, and experiences and perceptions of paternity leave. Our interview guide for company supervisors included questions on age, occupation, education, children, and opinions about paternity leave. All participants agreed to video-record the interview (except for one company supervisor, who participated only by voice because a video camera was not available).

The Research Ethics Committee of Tokyo Medical and Dental University (Institute of Science Tokyo) approved this study (M2021-128). All the participants provided written informed consent. Each interview lasted for approximately 60 min. Before transcribing the data, the participants were anonymised to protect their privacy. The first author transcribed the interviews verbatim and typed the notes in Japanese.

### Data analysis

Reflexive thematic analysis (TA) was adapted to analyze the data from the fathers and supervisors at companies (19–21). In reflexive TA, themes are generated “at the intersection of data, analytic process and subjectivity” (19–21). It integrates the research question, the authors’ knowledge, perspectives, and experiences, alongside the content of the data, to generate themes with validation (21). This method was selected because the first author was the father of a one-year-old infant and a regular employee of a private company. These factors made reflexive TA suitable, as it enabled a deeper exploration of the participants’ experiences. The final theme list was refined through an iterative process of merging and revising the codes, facilitated by discussions between NN and NM. We followed the Standards for Reporting Qualitative Research (22) in reporting this study. The first author performed thematic coding and developed a codebook in English with the second author (NN). Deductive coding was conducted based on a previous study on paternity leave (10), followed by inductive coding and modification of the initial codes. The final codes were organised based on the social-ecological model (8). ATLAS.ti (version 9) was used for data analysis.

We considered reflexivity throughout data collection and analyses (23). The first author's personal circumstances—being a student, the father of a one-year-old infant, and a full-time employee at a private company—may have influenced the research process. Living in an urban area with a spouse and child as a nuclear family without co-residing grandparents, the first author's family did not rely on multigenerational support, and the child was in good health. These factors likely influenced the study in three key areas: research design, data collection, and analysis. In terms of research design, the absence of co-residing grandparents may have limited the first author's awareness of how family-based social support, including that provided by grandparents, impacts parental leave decisions. Similarly, as a salaried employee in a corporate environment, the focus may have leaned toward workplace policies, potentially overlooking the unique challenges faced by self-employed or informal workers. Nevertheless, the study design, coinciding with the first author's own eligibility for childcare leave, may have brought nuanced insights into contemporary Japanese parental leave policies. For data collection, conducting interviews when the first author's child was over one year old may have shifted the focus toward post-birth parenting experiences, with less emphasis on pre-birth decisions. However, the first author's firsthand experience likely facilitated deeper and more empathetic engagement with participants, enriching the inquiry into parental leave practices. Finally, regarding analysis, the first author's active parenting responsibilities may have heightened sensitivity to themes related to childcare and parenting, while potentially underemphasizing broader aspects such as workplace advancement or personal leisure.

To separate the experiences of the author from those of the participants and minimize interpretation, we asked them additional questions whenever information was omitted from their answers. Furthermore, we explained that the participants could freely state their opinions because the interview data were anonymised before transcription. This approach was employed to ensure that the participants avoided criticism from family members or employers. Additionally, we attempted to minimise our interpretation during coding.

## Results

### Findings from father participants (FP)

Table 1 outlines the characteristics of the 21 fathers. Most (71%) had only children. Nearly half (48%) of the participants were postgraduate students. Slightly below half (43%) had finished or continued to attain a paternity leave of six days or more.

**Table 1:** Characteristics of father participants (*N* = 21).

		Number of fathers (*N* = 21)	%
Sex	Male	21	100%
	Female	0	0%
	Other	0	0%
Age (years)	25–29	1	5%
	30–34	12	57%
	35–39	7	33%
	40–44	1	5%
Number of children	1	15	71%
	2	6	29%
Age of youngest child (months)	0–3	5	24%
	4–7	13	62%
	8–11	3	14%
Marital status	Not married	0	0%
	Married/De facto marriage	21	100%
Education	Junior college	1	5%
	Graduate	9	43%
	Postgraduate	10	48%
	Not reported	1	5%
Occupation	Regular employee	17	81%
	Self-employed	3	14%
	Not reported	1	5%
Duration of paternity leave (day)	0	9	43%
	1–5	3	14%
	6–14	1	5%
	15–30	3	14%
	31–90	3	14%
	91–200	2	10%

### Themes associated with taking paternity leave

We organised the themes into four levels of the social-ecological model: individual, relationship, community, and societal (refer to Table 2) (8).

**Table 2:** Themes associated with taking paternity leave.

Level	Theme
Individual	Fathers’ career aspirations
	Fathers’ commitment to supporting infants or mothers
Relationship	Mothers’ or infants’ need for fathers’ involvement
Community	Colleagues’ outlook on paternity leaves
	Fathers’ work schedules or responsibilities
	Support from relatives
Societal	Paternity leave policies
	Trends toward fathers’ involvement

### Individual level

*Fathers’ career aspirations:* Some fathers mentioned career aspirations as a theme associated with attainment or length of paternity leave. They perceived that paternity leave would affect their promotion, continuation of their current occupational roles, and competition with colleagues. A respondent noted a barrier to taking paternity leave,

“No other colleagues took paternity leave; I guess I am the first case in my department… The more I get involved in childcare, the harder the competition with those who can work full-time with less time for childcare will be.” [FP1, age 35, two children, three-month paternity leave]

In contrast, a father referred to the positive aspect of paternity leave for career development by stating that childcare can support the career by providing experiences that fathers do not get from the work though he had no experience of taking paternity leave.

“If you practice childcare and understand how hard it is, then you will be able to make various considerations for those who are raising children when you work with them…getting involved in childcare will bring a plus to your experience and work. You will acquire experiences that do not come from work.” [FP7, age 36, two children, no paternity leave]

*Fathers’ commitment to supporting infants or mothers.* Most fathers suggested that their individual commitment to supporting infants or mothers was a reason for taking paternity leave. They had taken paternity leave because of their ideological commitment as fathers to raise infants or as husbands to support their partners. Regarding commitment to raising infants, one father noted,

“It is natural that both parents take care of infants… Paternity leave is necessary to witness, rather, experience the growth of infants.” [FP12, age 30, one child, no paternity leave]

As for support for mothers, one father said,

“We have a lot to learn while raising the first child. Mothers, too. It takes a long time to complete anything. Thus, mothers have a difficult time both physically and psychologically. On such occasions, mothers need the support of fathers.” [FP11, age 32, one child, no paternity leave]

### Relationship

*Mothers’ or infants’ need for fathers’ involvement.* Almost all fathers reported that they considered taking paternity leave in response to the mothers’ support needs, requests, or infants’ care needs. Regarding mothers’ support needs, fathers noted that their partners required physical or mental support after giving birth, which encouraged them to take the paternity leave.

“My wife gave birth by caesarean section. The following month, she was too sick to move around. I took paternity leave to support her.” [FP3, age 40, one child, three-month paternity leave]

“My wife said she was getting mad at our baby even though the little baby would not understand the anger. It was unlikely, but I feared that she could have been violent. I thought about this [her use of violence] a little and took paternity leave.” [FP6, age 30, two children, two-day paternity leave]

Some fathers said that their wives requested them to take the paternity leave, or that their needs motivated them to do so.

“I decided to take another paternity leave because my wife asked me to take off work on that day.” [FP15, age 38, two children, one-month paternity leave]

“For a few days, my baby had a fever.” [FP21, age 36, one child, 17-day paternity leave]

#### Community

*Colleagues’ outlook on paternity leaves.* Most fathers reported that their colleagues’ positive or negative attitudes toward paternity leave influenced their decision to take paternity leave. One father noted,

“My company announced that they recommended paternity leave. In fact, the staff did not think this way. There was a gap.” [FP6]

Another stated,

“In my district, the manager, who is female, was positive about my paternity leave. My fellows said that it was okay to take paternity leave, but I heard of an example where a boss asked to make it shorter. Therefore, it depends on the thoughts of the boss.” [FP18, age 29, one child, seven-month paternity leave]

*Fathers’ work schedules or responsibilities.* Almost all fathers perceived that working time, work schedule flexibility, work location, or individual responsibility influenced paternity leave. A father explained that he was worried about the responsibilities that would accumulate during his absence, which prevented him from taking paternity leave.

“If I had taken one or two weeks of paternity leave, my work after the leave would have been too much. I considered this, thought that paternity leave is unrealistic, and missed it.” [FP2, age 34, one child, no paternity leave]

Another father mentioned that, since he was working from home, he did not assume it was necessary for him to take paternity leave.

“In terms of the way I work, I became completely remote because of Corona [COVID-19]. I was able to stay at home, comparatively speaking, during my wife’s pregnancy, or even after the birth, well, I think I was able to be there for her all the time.” “Working from home every day was a significant factor. As I was always working from home, I did not feel it was absolutely necessary to take paternity leave.” [FP16, age 32, one child, no paternity leave]

*Support from relatives.* Some fathers explained that support for childcare from grandparents influenced their decision to take paternity leave. When couples received childcare support from relatives, especially grandparents, the mothers’ or infants’ need for the fathers’ involvement (at the relationship level) was reduced. One father noted,

“My wife’s mother was very supportive. She reduced her work time and was always with my baby. My wife told me that she was all right. We agree that I would focus on doing a good job at work.” [FP11]

#### Societal

*Paternity leave policies.* Some fathers stated that government policies influenced their decision to take paternity leave, particularly for those in regular employment. One father said,

“I make it a rule to take advantage of all the available programs.” [FP4, age 30, one child, five-day paternity leave]

On the contrary, one father who was not a regular employee (a freelance lawyer) had no access to such benefits. One father said,

“I am a lawyer. Most of us do have no employment contracts. Therefore, we do not have annual or childcare leave. The benefits that come with employment contracts are not available to us.” [FP9, age 34, two children, no paternity leave]

Even for fathers in regular employment, taking paternity leave often relied on whether their work responsibilities could be transferred to colleagues (at the community level). Without such support, the existence of paternity leave policies alone does not ensure that fathers can take leave. A father stated that

“I am not against childcare leave per se, but I think that it depends on the environment, the work environment. So, unless there is an environment in which other people can do your work for you, this kind of childcare leave will not progress in the first place.” [FP5, age 37, one child, no paternity leave]

*Trends toward fathers’ involvement.* Some fathers perceived that social trends were associated with paternity leaves. One father noted that his commitment to supporting his infant (at the individual level) was influenced by cultural trends that emphasized fathers’ involvement in childcare.

“Well, in the past there probably wasn't anything like that at all, but nowadays it’s natural that both the father and the mother should be involved in childcare, and that’s the way the world is going, and I myself, well, naturally I wanted to be actively involved in childcare.” [FP14, age 32, one child, three-week paternity leave]

### Findings from company supervisor (CS) participants

Table 3 presents the characteristics of the company supervisors who participated. All were regular employees. The supervisors mentioned themes related to paternity leave from the broader perspective of long-term effects and impact on the organisation. First, all of them pointed out that the experience of taking paternity leave helps fathers improve work productivity after paternity leave. A company supervisor noted,

**Table 3:** Characteristics of company supervisor participants (*N* = 4).

		Number of company supervisors (*N* = 4)	%
Sex	Male	3	75%
	Female	1	25%
Age (years)	35–39	1	25%
	40–44	2	50%
	45–49	1	25%
Number of children	0	1	25%
	1	1	25%
	2	2	50%
Marital status	Not married	0	0%
	Married/De facto marriage	4	100%
Education	Graduate	2	50%
	Postgraduate	2	50%
Occupation	Regular employee	4	100%
	Self-employed	0	0%

“You will have to think about how to keep things going when you are away. You may think it is troublesome to examine what is important or not necessary when performing tasks routinely. [If you take paternity leave] You have the opportunity to streamline your work. You will recognise what you need to hand over [to your colleagues] or what tasks you need to ask somebody [to do]. These are the positive aspects of paternity leave.” [CS2, age 37, no child, married, graduate]

Another supervisor noted,

“If you do not take paternity leave, you may work feeling that your time with your child is limited, or you are unable to support the birth of your baby. Rather, taking paternity leave will improve your productivity and work performance after having time with your child as you would like without regret.” [CS4, age 40, one child, married, postgraduate]

Second, some company supervisors mentioned that paternity leave could improve mothers’ productivity. One of them noted,

“I hope that my female subordinates will recover well and return to work soon after maternity leave. If the partner is willing to cooperate in childcare, I can expect that they [female subordinates] will be physically and mentally healthier and return to work sooner. Additionally, children would catch a cold, and parents may need to suddenly take days off after maternity leave. On such occasions, fathers are likely to take days off if they [fathers] have taken paternity leave.” [CS1, age 45]

Another company supervisor stated,

“I understand that [the paternity leave of my female subordinates’ partners] keeps the performance of my [female] subordinates when they have some troubles with their children.” [CS4, age 40]

Third, one company supervisor mentioned that paternity leave attracts colleagues’ attention to their work-life balance. He stated,

“The colleagues…will take this opportunity to think again about their own lifestyle, rather than to support an employee whose partner is taking paternity leave. They recognise that they should change the way they work or live with their family members. They might think that they would like to take an annual leave or paternity leave and ask how to do so. In this way, paternity leaves influence them. Probably, it would be more influential if our female colleague’s partner takes paternity leave outside our company, rather than a male colleague taking paternity leave.” [CS3, age 41]

Regardless of the company supervisors’ perceptions, they did not proactively encourage fathers to take paternity leave. One of them said,

“Although I can find messages promoting paternity leave at the office, each supervisor is not proactively communicating about paternity leave on a regular basis. When I hear that someone’s baby is born, I talk to them. Then, I hear from new fathers that their baby is born, and they would like to take paternity leave. I answer that they can certainly take it. Basically, they can take paternity leave when they need.” [CS4]

## Discussion

In this study, we explored factors related to paternity leave from the father’s perspective. According to the fathers’ perceptions, the attainment of paternity leave was associated with their career aspirations, commitment to supporting infants or mothers, mothers’ or infants’ need for fathers’ involvement, colleagues' outlook on paternity leaves, fathers’ work schedules or responsibilities, support from relatives, paternity leave policies, and trends toward fathers’ involvement. The inhibitory influence of fathers’ career aspirations on paternity leave was reported in a previous study in Spain (24). Considering the long employee tenure and seniority-based wages in Japan, fathers may be particularly anxious about the negative impact that the attainment of paternity leave could have on their careers (25–27). However, to the best of our knowledge, the promoting influence of fathers’ career aspirations on paternity leave has not been reported. As inclusion in the workplace has been drawing the attention of researchers since 2010 (28, 29), they have only recently begun to consider the importance of paternity leave experiences. The fathers’ commitment to supporting infants or mothers is consistent with the findings of a previous study in the US (12). Mothers’ or infants’ need for fathers’ involvement has not been reported in previous studies, except for the finding of mothers’ employment in Spain (10). The absence of this evidence may be that ‘need’ changes depending on individual circumstances and is difficult to measure with one scale in quantitative studies. The findings related to colleagues’ outlook on paternity leave are consistent with a previous study in Japan (15). At workplaces in Japan, this issue can be prominent because employees fear of being seen as disloyal and incompetent when they take leaves (14, 30). Regarding fathers’ work schedules or responsibilities, work time and individual responsibilities have been reported in Spain (24). Working hours have been reported in the US as well (12). However, the work location in this theme (i.e., fathers’ work schedules or responsibilities) has not been reported in previous studies. This study might have captured the association between coronavirus disease 2019 (COVID-19) and subsequent work from home (31) with paternity leave. The respondents spontaneously mentioned the work-from-home dynamics due to COVID-19. Instead of the social-ecological model, application of other models such as the health belief model (32) could have resulted in other health-related themes. Support from relatives was consistent with previous studies (33). Paternity leave policies is consistent with previous studies in Finland and Sweden (34). The theme ‘trends toward fathers’ involvement’ has not been reported in previous studies because previous studies, such as Petts (12) might have tried to represent this theme with other variables, such as occupation or race.

This study suggests that paternity leave can be encouraged by changing modifiable factors as follows. At the individual level, fathers should be able to regard paternity leave as an opportunity to become experienced managers at workplaces that promote diversity and inclusion. Regarding fathers’ commitment, fathers can consider a paternity leave to materialise their commitment to infants or mothers. At the relationship level, mothers may be able to promote paternity leave by expressing their needs or describing the infants’ needs to fathers. At the community level, company colleagues who are going to be new fathers will need to arrange work schedules and responsibilities so that they are not overloaded with accumulated work after paternity leave. Considering the company supervisors’ statement that paternity leave could have positive effects at work and fathers’ statements that work schedules and responsibilities prevent paternity leave, company colleagues may have the rationale to improve their baseline work-life balance in the first place to promote this practice. Companies can share actual examples of paternity leave with new fathers to change fathers’ perceptions of colleagues’ outlook on paternity leave. Besides, fathers’ relatives can suggest paternity leave when they are unable to offer support for childcare. At the societal level, governments should expand the scope of paternity leave policies and help fathers recognise that they can take paternity leaves. In Japan, the amendment of Child Care and Family Care Leave Act in 2021 improved the flexibility of the leave schedule and mandated employers to inform expecting parents of the policy (35). However, those who are not enrolled in employment insurance, such as self-employed business owners, are still not eligible for the benefit payment during paternity leave (35). In addition, the media can contribute to promoting paternity leave by reporting social trends toward fathers’ involvement in childcare or housework.

Our study has several implications. At the individual level, fathers can be encouraged to view paternity leave as an opportunity to demonstrate leadership in workplaces that prioritize diversity and inclusion (36). Additionally, emphasizing how paternity leave enables fathers to actively commit to supporting both infants and mothers can further underscore its importance. At the relationship level, mothers can play a crucial role in fostering paternity leave uptake. They can achieve this by clearly communicating their own needs for paternity leave and emphasizing the needs of their infants, particularly by highlighting the positive impacts such leave can have on family well-being and child development. Additionally, engaging in open and honest discussions between mothers and fathers about fathers’ career aspirations and their responsibilities to support mothers and infants can foster mutual understanding. By sharing their perspectives candidly, fathers can gain a deeper appreciation of these needs, which may further motivate them to take paternity leave. At the community level, company supervisors can play a pivotal role in promoting paternity leave by providing tailored suggestions to fathers about how taking leave can align with and even accelerate their professional careers. Supervisors can also emphasize how taking paternity leave demonstrates fathers’ commitment to supporting their families. Supervisors can address fathers’ potential misperceptions about workplace attitudes. Research shows that fathers sometimes misjudge their colleagues’ perspectives on paternity leave (15). To address this, supervisors could initiate team discussions to make the team’s attitudes toward paternity leave more transparent (15). These efforts, along with proactive actions such as facilitating job redistribution and flexible scheduling to make paternity leave more feasible, could be formally recognized and incorporated into supervisors’ performance evaluations. Such measures would not only incentivize supervisors but also help establish paternity leave support as a shared organizational goal. Governments can encourage companies to involve fathers’ supervisors in the decision to take paternity leave so that fathers can learn to work with people from diverse backgrounds. Japan has implemented paternity leave policies that mandates companies to support paternity leave-taking in addition to working from home for new fathers (37). To ensure inclusivity, however, it would be more effective if governments took steps to promote paternity leave as a universal right, accessible to all fathers regardless of their employment status (38). This approach addresses concerns such as those raised by a self-employed respondent who remarked, “we do not have annual or childcare leave.” Furthermore, governments can enhance these efforts by publishing surveys and reports on companies’ paternity leave uptake and workplace attitudes toward leave (37). Such transparency could help mitigate the negative perceptions fathers may hold about their colleagues’ views on taking leave. Future quantitative research measuring the relative importance of each factor is needed to efficiently promote paternity leave. Further research should also be conducted to confirm whether career aspirations act as a barrier or a motivator for taking paternity leave. Possible intervention programs focusing on paternity leave will require further studies to evaluate its effectiveness. Interviewing other stakeholders, such as mothers and policymakers, should be beneficial in improving the environment for paternity leave.

### Study limitation

This study had several limitations. First, the first author, as a full-time employee at a private company and a postgraduate student, recruited participants primarily through personal networks. As a result, nearly half of the fathers were postgraduates, and most were regular employees. This recruitment approach may have not included perspectives from fathers with different occupational backgrounds, such as self-employed individuals, farmers, or freelancers, who may face unique challenges with childcare leave, including income loss or job insecurity. Furthermore, fathers with potentially higher childcare leave needs, such as those raising children with congenital conditions, may have also been underrepresented in the sample. Therefore, the results may not be transferable to fathers with characteristics substantially different from those of the study participants. Likewise, the new findings—the promoting influence of fathers’ career aspirations, mothers’ or infants’ need for fathers’ involvement, and fathers’ work location—may not be transferable to countries with low inclusiveness (39), wide economic gender gap (40), or poor digital adoption (41), respectively. Second, another limitation of the study was the small number of company supervisors included as key informants in the interviews (18). Third, the participating fathers might have avoided sensitive topics, such as medical history, sexual problems, or financial difficulties. However, these topics can fall into themes such as the mothers’ or infants’ need for fathers’ involvement or the paternity leave policies.

## Conclusions

This study revealed fathers’ perceptions regarding the factors associated with taking paternity leave. The four themes—“fathers’ career aspirations”, “mothers’ or infants’ need for fathers’ involvement”, “fathers’ work schedules or responsibilities”, and “trends toward fathers’ involvement”—expanded the existing evidence base. The results may provide useful information on how to create an environment wherein fathers can easily take this leave. Future quantitative research measuring the relative importance of each factor is needed to efficiently promote paternity leave. Possible intervention programs focusing on paternity leave will require further studies to evaluate their effectiveness.

## Data Availability

The original contributions presented in the study are included in the article/Supplementary Material, further inquiries can be directed to the corresponding author.
