# Supplementary material for: Fathers’ perceptions of factors associated with the attainment of paternity leave: a qualitative study
Source: Front Glob Womens Health. 2025 Jan 28;5:1466227. doi: 10.3389/fgwh.2024.1466227 (PMC11831607; doi:10.3389/fgwh.2024.1466227)
Supplement: Supplementary file 1 [file Supplementaryfile1.docx]

## **Supplementary Material. Interview Guide**

### **Guide 1. Interview Guide for Fathers**

Interview guide (for fathers)

Below, the instructions to the interviewer are in *italics.*

1. *Introduction*

Thank you very much for taking time out of your busy schedule to participate in our survey, ‘A Qualitative Study on the Influence of Paternity Leave on Mothers' Postpartum Depression’. My name is _______ and I am a co-investigator of this study at <anonymised for blind peer review> University.

1. *Confirmation of the interview environment and the background of the research participant (exclusion criteria)*

- **Are you currently participating in this interview in an environment where no one else, including your family, can hear what you are saying?**
- Are there any problems with the audio and video connections?
- **Do you have any health problems that would prevent you from participating in the one-hour online interview now?**

1. *Verification of the identity of the interviewee*

To conduct the research according to the prescribed procedure, please allow me to verify your identity. Please tell me your full name.

1. *Intent of the study*

In Japan, the proportion of fathers who take paternity leave after the birth of their babies is limited, but it is on the rise. The government is planning to increase the proportion by amending the law. Mothers sometimes experience postpartum depression after the birth of their babies, and further measures are needed. We are examining the impact of fathers taking paternity leave on preventing postpartum depression in mothers. Therefore, we would like to interview fathers who have babies or supervisors who have subordinates to ask about their perceptions and thoughts on paternity leave and mothers' postpartum depression.

1. *Confirmation of explanation and consent*
   1. Did you understand the explanation of this study that I sent you earlier?
   2. Do you have any questions about this study and the consent form?
   3. Are you still willing to participate?
       **<Send the consent form and obtain the signature>**
      1. If consented: Although you have given your consent now, if there is a question you do not want to answer during the interview, you can say ‘I do not want to answer’ and refrain from answering. Moreover, if you want to stop the interview or withdraw your consent, please let me know at any time.
2. *Start recording*

As mentioned in the explanation, we need to record this interview for the analysis of the study. May I begin now?

＜Start ZOOM recording>

＜Start voice recorder>

1. *Background of the interviewee*

Please allow me to confirm that you are eligible for this study. Moreover, please let me know your demographic information so that I can report the background of the people I interviewed when I report the results of this study. Please note that the results will be anonymised so that you will not be identified when we report the results. There is nothing wrong with any of your answers, so please be frank.

- **Sex (male/female)**
- **Age (answer in whole numbers)**
- **Have you continued to live in Japan for the past year? (yes or no)**
- **Is Japanese your first language?**
  - **If it is not your native language, do you have JLPT N2 or higher?**
- **Age of all dependent children (~months if under 1 year old)**
- Date of birth of youngest child (year, month)
- If the youngest child was born at hometown, the duration of being back at hometown (from birth to _ months)
- **Period of separation from the child's mother or the youngest child (including temporary separation owing to returning to hometown or hospitalization), if any, between the birth of the youngest child and this interview (~ months).**
- Number of days in the past month when you have received support from relatives, babysitters, etc. in raising your youngest child (~ days per month)
- If your youngest child attends a preschool, when did he/she entered the preschool (from Month, Year)?
- Marital status (married or de facto marriage, number of years)
- Educational background (junior high school, high school, vocational school, junior college, technical college, graduate, postgraduate, other)
- **Employment status (working, working but on leave (paternity leave, nursing care leave, etc.), not working)**
  - **Current**
  - **At birth of the youngest child**
- Type of job contract (executive officer at a company or other organisation, self-employed, help with family business, paid work at home, regular employee, part-time job, dispatched employee at a dispatch office, contract worker, part-time worker, other)
  - Current
  - At birth of the youngest child
- Type of work (administrative work, professional and technical work, clerical work, sales work, service work, security work, agriculture, forestry and fishery work, production process work, transportation machinery operation work, construction and mining work, transportation, cleaning and packaging work, other work)
  - Current
  - At birth of the youngest child
- **Whether or not a manager with subordinates**
  - Current
  - At birth of the youngest child
- If you have subordinates, do you keep track of their attendance?
  - Current
  - At birth of the youngest child

*If the participant does not meet the inclusion/exclusion criteria*

We have decided the conditions of the participants in this study, authored the research protocol, applied, and obtained the approval by the review committee. From what you have just told me, I know that you do not meet those conditions. I am afraid that we have to stop here. As you have spared the time for this study, we will send you the gift card later.

This is where I start asking about paternity leave and postpartum depression. Any opinions are valuable and never wrong, so please be frank with us.

1. *About Paternity Leave*
   I would like to ask about paternity leave. In this interview, please consider that ‘paternity leave’ refers not only to the narrow sense of the words, but also refers to any leave you take as a result of the birth of your child, including annual paid leave.
   1. How did you work just before your youngest child was born?
      *Probe:*Did you work from home? How many days per week?
      How much overtime did you work each month?
      Were you able to flexibly change the starting or closing time of your work schedule for personal reasons?
   2. What do you know about paternity leave?
      *Probe:*What do you know about the general situation of the attainment of paternity leave?
      What do you know about the paternity leave system?
      Do you have any impressions about fathers taking paternity leave?
      What do you think are the reasons why fathers need to take paternity leave?
      How do you think paternity leave relates to the new baby?
      How do you think paternity leave will affect the mother?
   3. Have you ever taken paternity leave? (Please indicate the number of days from attending the childbirth to the present.)
      1. Please tell me the duration (days, months) *If the father has taken paternity leave*
      2. Which type of leave did you use? *If the father has taken paternity leave
         Probe:*Did you use any other types of leave? Statutory leave includes annual paid leave, paternity leave, child nursing and nursing care leave, and special leave includes congratulation or condolence leave, refreshing leave, and summer and winter vacations.
   4. How did you decide to take/not to take ~ days of paternity leave?
      *Probe:*Did you have a chance to think about whether or not to take paternity leave?
      When did you decide whether or not to take paternity leave?
      When you decided how to take paternity leave, did you communicate with your supervisor, coworkers, family, or friends about it?
      What did you take into consideration when deciding whether or not to take paternity leave?
      What do you think were **the reasons for your decision whether or not to take paternity leave?**
      What factors did you consider when deciding on the length and timing of your paternity leave?
      What do you think were **the reasons for your decision on the length and timing of your paternity leave?**Is that reason related to the way you worked before your child was born?
      In addition to what you have answered so far, were there any other reasons, circumstances, or feelings that influenced your decision to take or not take paternity leave?
2. Paternity leave and maternal postpartum depression
   I would like to ask you about paternity leave and maternal postpartum depression.
   1. What changes have occurred in yourself since the birth of your youngest child?
      *Probe:*Did you experience any changes with your partner, the mother?
      Before the birth of your youngest child, what role of the father did you anticipate?
      After the birth of your youngest child, did your role as a father differ from your prenatal expectations?
      Is there anything you have done for your family?
      Have there been any changes in your emotional state?
   2. What do you know about maternal postpartum depression?
      *Probe:*Under what conditions do you think mothers of infants are more likely to suffer from postpartum depression?
   3. The general symptoms of postpartum depression in mothers are said to be as shown in <Table 1>. In addition, <Table 2> shows the factors of fathers that have been examined in relation to postpartum depression in mothers.
       *Show below on the screen:*
      **Table 1: Common symptoms of postpartum depression^[[1]](#footnote-1)^**Sleep disturbance (not limited to the insufficient sleep owing to childcare)
      Feels strong anxiety
      Reacts easily to small things and has a short temper
      Mentally overwhelmed and unable to handle things
      Obsessive about the baby’s health and diet

      **Table 2 Fathers’ factors being examined for association with postpartum depression in mothers^[[2]](#footnote-2)^**
      <Fathers themselves>
      Amount and frequency of alcohol and tobacco use
      Father’s stress (degree to which father’s stress affects mother)
      <Marital relationship>
      Whether or not the couple can and does communicate with each other
      Frequency of disagreements between husband and wife and degree to which they hurt each other verbally
      The degree to which couples control each other (make important decisions on their own without consulting each other).
      Emotional intimacy (mutual trust, affection, and commitment to the relationship)
      Emotional support (acknowledging the other person’s feelings, perceptions, and behaviours, and demonstrating the understanding and acceptance)
      Satisfaction with the marital relationship
      Sexual satisfaction
      Non-cooperation and absence (treating mother coldly, not being there when support is needed)
      <Housework, childcare, etc.>
      Degree to which housework is shared at a satisfactory ratio
      Overall evaluation of support (degree of fathers being dependable)
      Provision of information (provision of knowledge that leads to problem-solving)
      Tangible support (helping with childcare, housework, etc.)
      Father cares for the baby alone (daytime or night-time)
      Taking the baby to the doctor
      1. Is there anything that you have done to reduce the possibility of maternal postpartum depression? Please do not limit your answer to the things listed in Tables 1 and 2, and include things that are not directly related to paternity leave.
         *Probe:*Is there anything that you did by intention?
          Something related to the mother’s life
          Something directly related to childcare
          Something related to the home environment
          Something affecting the relationship among family members
          Other things
         Is there anything that you did not intend to do at the time, but in retrospect you think would have reduced the possibility of postpartum depression?
         Is there anything you did to mitigate rather than prevent maternal postpartum depression?
         How did you feel when you were doing the things you just mentioned?
      2. What changes for mothers do you think the things you have mentioned so far have made?
         *Probe:*
         Did you notice anything by yourself?
         Were there anything the mother said to you?
         Are there any changes related to postpartum depression that you tried to bring about but could not?
         Are there any changes related to postpartum depression that you wish you could have made in retrospect?
      3. Of the things you have answered so far, have any of them come true because you took paternity leave?  *If the respondent has taken paternity leave, ask about their answers to 8.1 and 8.3.1
         Probe:*
         What changes has paternity leave brought about that have enabled you to do this?
         Is there anything that you were able to do more because of your paternity leave?
         Is there anything you have learned as a result of your paternity leave?
      4. Is there anything that you wish you could have done had you taken paternity leave differently?  *If the respondent has taken paternity leave, ask about their answers to 8.1 and 8.3.1*
         *Probe:*Assume that you took paternity leave longer than you actually did
         Assume that you took paternity leave at a different time
      5. Is there anything you have answered so far that you think you would have achieved if you had taken paternity leave?  *If the respondent* ***did not take*** *paternity leave, ask about the answers to 8.1 and 8.3.1.*
3. *Supplementary information*

These are the questions that I prepared in advance. Is there anything that you didn’t have time to answer during the course and would like to add?

We will now end the recording.

1. *Request for referral*

In this study, we are using the method of asking interviewees to introduce the next respondent. Do you know of anyone who meets the requirements on the screen (*display consent document on the screen*) who might be interested in participating in this interview? If so, can we ask you to obtain their consent and share the contact information with me?

1. *Closing*

Lastly, do you have any questions? If you have any, please don’t hesitate to ask.

As we proceed with the analysis, may we contact you again if we have any additional questions? Moreover, when the results are settled to some extent, we would like to have an opportunity to confirm whether the results are convincing or not if possible.

Thank you very much for your time today.

### **Guide 2. Interview Guide for Company Supervisors**

Interview guide (for company supervisors)

The instructions to the interviewer are in *italics.*

1. *Introduction*

Thank you very much for taking time out of your busy schedule to meet with us today for our survey, ‘A Qualitative Study on the Influence of Paternity Leave on Mothers’ Postpartum Depression’. My name is _______ and I am a co-investigator of this study at <anonymised for blind peer review> University.

1. *Confirmation of the interview environment and the background of research participant (exclusion criteria)*

- Are you currently participating in this interview in an environment where no one else, including your family, can hear what you are saying?
- Are there any problems with the audio and video connections?
- Do you have any health problems that would prevent you from participating in the one-hour online interview now?

1. *Verification of the identity of the interviewee*

In order to conduct the research according to the prescribed procedures, please allow me to verify your identity. Please tell me your full name.

1. *Intent of the study*

In Japan, the proportion of fathers who take paternity leave after the birth of their babies is limited, but it is on the rise. The government is planning to increase the proportion by amending the law. Mothers sometimes experience postpartum depression after the birth of their babies, and further measures are needed. We are examining the impact of fathers taking paternity leave on preventing postpartum depression in mothers. Therefore, we would like to interview fathers who have babies or supervisors who have subordinates to ask about their perceptions and thoughts on paternity leave and mothers’ postpartum depression.

1. *Confirmation of explanation and consent*
   1. Did you understand the explanation of this study that I sent you earlier?
   2. Do you have any questions about this study and the consent form?
   3. Are you still willing to participate?
      1. If consented: Although you have given your consent now, if there is a question you do not want to answer during the interview, you can say ‘I do not want to answer’ and refrain from answering. Moreover, if you want to stop the interview or withdraw your consent, please let me know at any time.
2. *Start recording*

As mentioned in the explanation, we need to record this interview for the analysis of the study. May I begin now?

1. *Background of the interviewee*

Please allow me to confirm that you are the eligible for this study. Moreover, please let me know your demographic information so that I can report the background of the people I interviewed when I report the results of this study. Please note that your background information will not be used to identify you personally when we report the results.

- Sex (male/female)
- Age (answer in whole numbers)
- Have you continued to live in Japan for the past year? (yes or no)
- Is Japanese your first language?
  - If it is not your native language, do you have JLPT N2 or higher?
- Age of all dependent children **(~months if under 1 year old)**
- Date of birth of youngest child (year, month)
- Marital status (married or de facto marriage, number of years)
- Educational background (junior high school, high school, vocational school, junior college, technical college, graduate, postgraduate, other)
- Current employment status (working, working but on leave (paternity leave, nursing care leave, etc.), not working)
- Current type of job contract (executive officer at a company or other organisation, self-employed, help with family business, paid work at home, regular employee, part-time job, dispatched employee at a dispatch office, contract worker, part-time worker, other)
- Current type of work (administrative work, professional and technical work, clerical work, sales work, service work, security work, agriculture, forestry and fishing work, production process work, transportation machinery operation work, construction and mining work, transportation, cleaning and packaging work, other work)
- Whether or not you are currently a manager with subordinates.
  - Are you currently managing the attendance of your subordinates?

*If the participant does not meet the inclusion/exclusion criteria*

We have decided the conditions of the participants in this study, authored the research protocol, applied, and obtained the approval by the review committee. From what you have just told me, I know that you do not meet those conditions. I am afraid that we have to stop here. As you have spared the time for this study, we will send you the gift card later.

This is where I start asking about paternity leave and postpartum depression. Any opinions are valuable and never wrong, so please be frank with us.

1. *About Paternity Leave*I would like to ask about paternity leave. In this interview, please consider that ‘paternity leave’ refers not only to the narrow sense of the words, but also refers to any leave you take as a result of the birth of your child, including annual paid leave.
   1. Have any of your male subordinates ever taken paternity leave?

*Probe:*
Have any of your subordinates ever had children?

- - 1. Please tell me the duration of the paternity leave. (~days, ~months) *If there was a case*
    2. Which type of leave was used? *If there was a case
       Probe:*Were any other types of leave used? Statutory leave includes annual paid leave, paternity leave, child nursing and nursing care leave, and special leave includes congratulation or condolence leave, refreshing leave, and summer and winter vacations.
  1. What do you know about paternity leave?
     *Probe:*What do you know about the general situation of the attainment of paternity leave?
     What do you know about the paternity leave system?
     Do you have any impressions about fathers taking paternity leave?
     What do you think are the reasons why fathers need to take paternity leave?
     How do you think it relates to the new baby?
     How do you think paternity leave will affect the mother?
  2. Have you ever talked about paternity leave with your male or female subordinates?
     1. What was the topic*? If the interviewee has talked about it.*
  3. How do you think the decision for fathers to take/not to take paternity leave is made in your workplace?
     *Probe:*Is it prescribed to set aside time to discuss whether or not to take paternity leave?
     Is the timing to decide whether or not to take paternity leave defined?
     Who are involved in the decision to take paternity leave?
     What reasons do you think make fathers decide whether to take paternity leave?
     What reasons do you think will determine the length and timing of paternity leave?
     Is it related to the way fathers worked before your child was born?
     In addition to what you have answered so far, do you think there are any other reasons, circumstances, or feelings that might affect the decision to take or not take paternity leave?
     1. As a supervisor, how would you be involved in the decision?
        *Probe:*How would you respond if your subordinates ask for your opinion on whether they should take maternity leave?
        How would you respond if your subordinates ask for your opinion on the length and timing of paternity leave?
        What do you consider when you do so?
        Even if you can't actually speak up in front of your subordinates, is there anything you would consider?
     2. What do you think your male subordinate himself would consider?
  4. How do you feel about your male subordinates taking paternity leave?
     *Probe:*
     What reasons, if any, do you have for encouraging them to take paternity leave?
     What reasons, if any, would you give for not recommending it?
     What do you think other people in the company would think about it?
     1. How do you think your workplace will be affected if your male subordinate takes paternity leave?
     2. How do you think your male subordinate will be affected if he takes paternity leave?
     3. Do you know of any actual cases of such an effect?
     4. Do you think there is a relationship between paternity leave and maternal postpartum depression?

1. Paternity leave of male subordinates and postpartum depression of their partners (mothers)
   We would now like to ask you about paternity leave and their partners' (mothers') postpartum depression.
   1. If your male subordinate has a child, what changes would you expect to see in him?
      *Probe:*
      What role do you think he will play as a father?
      Is there anything he would do directly for the child?
      What would he do directly for the mother (subordinate’s partner)?
      What do you think he will do for his family?
      What changes do you expect in his feelings?
      What problems do you think he will face?
   2. What do you know about maternal postpartum depression?
      *Probe:*Under what conditions do you think mothers of babies are more likely to suffer from postpartum depression?
   3. The general symptoms of postpartum depression in mothers are said to be as shown in <Table 1>. In addition, <Table 2> shows the factors of fathers that have been examined in relation to postpartum depression in mothers.
       *Show below on the screen*
      **Table 1: Common symptoms of postpartum depression ^[[3]](#footnote-3)^**Sleep disturbance (not limited to the insufficient sleep owing to childcare)
      Feel strong anxiety
      Reacts easily to small things and has a short temper
      Mentally overwhelmed and unable to handle things
      Obsessive about the baby's health and diet

      **Table 2 Fathers' factors being examined for association with postpartum depression in mothers ^[[4]](#footnote-4)^**
      <Fathers themselves>
      Amount and frequency of alcohol and tobacco use
      Father's stress (degree to which father's stress affects mother)
      <Marital relationship>
      Whether or not the couple can and does communicate with each other
      Frequency of disagreements between husband and wife and degree to which they hurt each other verbally
      The degree to which couples control each other (make important decisions on their own without consulting each other).
      Emotional intimacy (mutual trust, affection, and commitment to the relationship)
      Emotional support (acknowledging the other person's feelings, perceptions, and behaviours, and demonstrating the understanding and acceptance)
      Satisfaction with the marital relationship
      Sexual satisfaction
      Non-cooperation and absence (treating mother coldly, not being there when support is needed)
      <Housework, childcare, etc.>
      Degree to which housework is shared at a satisfactory ratio
      Overall evaluation of support (degree of fathers being dependable)
      Provision of information (provision of knowledge that leads to problem solving)
      Tangible support (helping with childcare, housework, etc.)
      Father cares for the baby alone (daytime or night-time)
      Taking the baby to see the doctor
      1. Are there any things that you would expect fathers do, intentionally or unintentionally, to reduce the likelihood of postpartum depression in mothers? (Please do not limit your answer to the things listed in Tables 1 and 2, and include things that are not directly related to paternity leave.
         *Probe:*
         Something related to the mother's life
         Something directly related to childcare
         Something related to the home environment
         Something affecting the relationship among family members
         Other things
         Is there anything you expect fathers do to mitigate the symptoms of postpartum depression in mothers rather than to prevent it?
      2. What changes for mothers do you think the things you have mentioned so far will make?
      3. What are things that fathers do to reduce the likelihood of postpartum depression in mothers that they might not be able to do without paternity leave?
         *Probe:*Is there anything they could do by taking a longer paternity leave?
         Is there anything they could do if they take paternity leave at a different time?
   4. If your male subordinate takes paternity leave, how do you think it will influence his partner (mother)?
   5. When you have a female subordinate who is giving birth, how do you feel about her partner (father) taking paternity leave?
      *Probe:*Would you like your female subordinate's partner to take paternity leave?
      Do you think it is realistic for your female subordinate's partner to take paternity leave? How do you think it will influence the mother if the father takes paternity leave?
      If your female subordinate's partner takes paternity leave, do you think it will have an impact on your department?
2. *Supplementary information*

These are the questions that I prepared in advance. Is there anything that you didn't have time to answer during the course and would like to add?

We will now end the recording.

1. *Request for referral*

In this study, we are using the method of asking interviewees to introduce the next respondent. Do you know of anyone who meets the requirements on the screen (*display consent document on the screen)* who might be interested in participating in this interview? If so, can we ask you to obtain their consent and share the contact information with me?

1. *Closing*

Lastly, do you have any questions? If you have any, please don’t hesitate to ask.

As we proceed with the analysis, may we contact you again if we have any additional questions? Moreover, when the results are settled to some extent, we would like to have an opportunity to confirm whether the analysis results are convincing or not if possible.

Thank you very much for your time today.

1. Stewart DE, Vigod S. Postpartum Depression. N Engl J Med. 2016 Dec 1;375(22):2177-2186. doi: 10.1056/NEJMcp1607649 [↑](#footnote-ref-1)
2. Pilkington PD, Milne LC, Cairns KE, Lewis J, Whelan TA. Modifiable partner factors associated with perinatal depression and anxiety: a systematic J Affect Disord. 2015 Jun 1;178:165-80. doi: 10.1016/j.jad.2015.02.023. Epub 2015 Mar 6. PMID: 25837550.

   N. Séjourné, V. Vaslot, M. Beaumé, N. Goutaudier & H. Chabrol (2012) The impact of paternity leave and paternal involvement in child care on maternal postpartum depression, Journal of Reproductive and Infant Psychology, 30:2, 135-144, DOI: 10.1080/02646838.2012.693155 [↑](#footnote-ref-2)
3. Stewart DE, Vigod S. Postpartum Depression. N Engl J Med. 2016 Dec 1;375(22):2177-2186. doi: 10.1056/NEJMcp1607649 [↑](#footnote-ref-3)
4. Pilkington PD, Milne LC, Cairns KE, Lewis J, Whelan TA. Modifiable partner factors associated with perinatal depression and anxiety: a systematic J Affect Disord. 2015 Jun 1;178:165-80. doi: 10.1016/j.jad.2015.02.023. Epub 2015 Mar 6. PMID: 25837550.

   N. Séjourné, V. Vaslot, M. Beaumé, N. Goutaudier & H. Chabrol (2012) The impact of paternity leave and paternal involvement in child care on maternal postpartum depression, Journal of Reproductive and Infant Psychology, 30:2, 135-144, DOI: 10.1080/02646838.2012.693155 [↑](#footnote-ref-4)
